# Annotations

**Published:** 1899-08-19

**Authors:** 


### Annotations.

### The Plague.

Several letters have lately appeared in the lay press in regard to plague, the writers of which, basing their arguments chiefly on the history of epidemics in the past, urge the possibility and even the probability of plague reaching England. We admit the possibility; indeed, we will go further, and say that the ascertained connection of the disease with conditions of dirt, overcrowding, and innutrition, such as, we fear, are not uncommon in some parts of our greatest cities, renders it probable that if the disease were once to obtain a foothold in some of our slums it might be a very difficult matter indeed to dislodge it. On the other hand, there are many reasons which may properly lead to a considerable amount of confidence in our power to resist such an incursion. Many years have passed away, and much has happened since Sir John Simon first expressed his belief that, as one result of increasing amount and rapidity of intercourse, "epidemics current in India might become current also in England," Since then the sanitary condition and administration of this country have been revolutionised; new defences against the introduction of infectious disease have been established, and much has been done to improve the surroundings amid which the people live and work. To take a single but very important difference between the London of 1666 and the London of 1899, the improved drainage has to a large degree eliminated the rats from our houses, and rats are known not only to breed the plague bacilli in incalculable numbers, but also to furnish a principal means of their conveyance from place to place. Neither have we, even in the worst slums of our great towns, anything approaching to the dirt of poor oriental dwellings. Our population, again, even in its lowest strata, is better fed and more resisting than that of Bombay or of Alexandria. Thus we may with considerable confidence, hope for the best. Moreover, history cuts two ways, for while it may be taken as showing the tendency of plague to spread in certain seasons, and as suggesting that having got so far as Alexandria it may well advance as far as England, it also teaches with great plainness that plague has again and again existed with far greater virulence than at present in Egypt, in Persia, in Syria, and even in the South of Russia, without showing any sign of being able to advance westwards toward this country. So far as the history of the last two hundred years is concerned, the argument is all against our being liable to suffer from plague. Unfortunately, history is not everything. Modern methods of travel and modern rapidity of communication between distant countries have introduced new conditions as to the operation of which history tells us nothing, and the confirmation received this week of the rumour which has been current for some time as to the existence of plague at Oporto is a matter of serious moment. By itself it would mean but little, but taken in conjunction with the very remarkable history of plague during the past five or six years it may mean a good deal. If the recent prevalence of this disease had merely been characterised by the occurrence of a larger number of cases than ordinary in the places which are usually visited by it there would be nothing to cause anxiety. But that is not so. Something has happened—it is hard to say what—by which the course of plague has been turned into new tracks, and it would be idle to pretend that its steady advance westwards, of which the outbreak at Oporto is the most recent sign, is to be regarded without mistrust.

### The late Sir Edward Frankland.

We regret to have to record the death of Sir Edward Frankland, who, although known to the present generation chiefly in connection with the analyses and reports on the character of the London water which he has made regularly month by month for more than thirty years, had in the earlier part of his career occupied an almost unique position among those who were then engaged in laying the foundations on which many of our modern conceptions of organic chemistry are founded. He was born in the year 1825, and ever since his student days he has been actively engaged in what even to the end may be termed original work. His has indeed been a long and honourable career, for it must have fallen to the lot of but few to devote so long a series of years to work of such utility, or to have been able to throw light on such a wide variety of subjects as have been dealt with, at one time or another, by Sir Edward Frankland. Although for many years past his position in the scientific world has been chiefly that of a specialist on water, specialism in his case did not mean, as we are afraid it does with too many, that he only knew one thing, but rather that he had concentrated on one group of subjects a very wide range of knowledge and of research. We need not attempt to hide the fact that in the death of Sir Edward Frankland the Thames as a source of water supply has lost one of its most important champions. The history of Sir Edward Frankland's official connection with the investigation of the metropolitan

ANNOTATIONS—*continued.*

water supply would form an interesting chapter in the history of the means which have been adopted for the provision of pure water in a thickly-populated country. He began by doubting greatly the possibility of ever restoring to purity water which had once been so defiled as the Thames is in its course, but his prolonged observation of the marvellous effects which can be produced by sand filtration led him of late years to declare unequivocally in favour of the Thames as a source from which, by aid of proper storage and filtration, it would be possible to obtain an ample supply of wholesome drinking water.

### The Emigration of Pauper Children.

The State Children's Association has addressed a memorial to the President of the Local Government Board urging the desirability of encouraging the emigration of suitable children, under the control of boards of guardians, in accordance with the conditions of Acts passed in 1897 in Ontario and Manitoba to regulate the immigration of certain classes of children. The emigration committee of the association have made enquiries into the working of those voluntary societies who send out children to Canada, and they are convinced that, if carefully carried out, emigration is one of the most wholesome and economical ways of dealing with State-dependent children. They now go so far as to urge the relaxation of the rule of the Local Government Board which forbids the emigation of girls over twelve years of age, experience having shown, they say, that with habits and minds well formed in an English training home such girls do admirably well. With much of this we can fully agree. Only the very fringe of the problem, however, is touched by the suggestion. "Selected" girls, "with habits and minds well formed," who have been taught the elements of "ordinary domestic work," certainly need not do ill in England. The difficulty comes with others, and we are afraid that these others the colonies will be somewhat loth to accept.

### The Teaching of Pharmacology.

A time may come when the reign of drugs shall cease, but that time is not yet, nor will it be in our day, and it may be broadly stated that any man who affects to despise drugs in the treatment of disease is not only throwing away one of the most potent means for the relief of his patients, but is drawing contempt upon his profession, for whether we like it or not the life of the physician must be largely passed in the ordering of drugs, and in fitting them as accurately as his knowledge will allow to the ailments of his patients. His knowledge of this subject should then be as wide as possible, and we are sure that Professor Bradbury does well in urging as he does that pharmacology should be thoroughly studied by every medical student, and that a knowledge of the Pharmacopœia should be made a compulsory part of his curriculum. "Think for a moment," he says, "of what is required of a medical man once started in practice. A considerable part of his work is the writing of prescriptions for his patients, and yet in respect of the knowledge of drugs and their actions I maintain his education is often most imperfect. . . . Is it too much to expect those who are daily to prescribe remedies to be acquainted with what is known of their actions? Certainly not. . . . The more intimately a student knows the action of the drugs he prescribes the greater will be his success in treatment, and it is much to be regretted that some of the examining boards require a student to acquire this knowledge only in a haphazard way, or at a premature stage of his course." Professor Bradbury goes on to say that the student cannot appreciate the physiological action of drugs until he knows the elements at least of physiology, while on the other hand he cannot appreciate the importance of judicious prescribing unless he has learned the nature and properties of the agents prescribed. He would therefore so arrange the position of pharmacology in the curriculum that it should come after anatomy and physiology, and alongside of pathology. No doubt the medical student of to-day is already vastly overladen with subjects, but we think that no one can gainsay Professor Bradbury's contention, that so long as a main part of a doctor's work consists of the prescribing of drugs he should know as much as possible about their actions.

### What is a Common Lodging House?

The decision recently given at the South-Western Police-court to the effect that the Rowton Houses are not to be regarded as common lodging houses is one which seems to be in accordance both with facts and common sense, and, so far as it concerns the particular case in question, is one which is not likely to be attended with any evil consequences. There is, however, considerable danger in all decisions which tend to whittle away the stringency of the Common Lodging Houses Act, and the danger in this case arises from the use which is likely to be made of the decision by those who wish to evade the Act. If the so-called "shelters" are to be free from inspection because they are charities, and other buildings because they are not, in the words of the magistrate, "open to all comers," and others because food is supplied according to a tariff instead of being brought in by the inmates and cooked at the common kitchen fire, it will be possible to evade the Act in every direction, and to establish once again the very sort of dens for the suppression of which legislation was undertaken—dens which will be unwholesome, both from the moral and the hygienic point of view, and will be independent of police inspection as well as of all sanitary supervision.

### Non-Nitrogenous Diet in Epilepsy.

In the treatment of epilepsy Dr. Haig advises the employment of a non-nitrogenous diet. He recommends the total disuse of meat, eggs, and tea, with the object of providing a diet free from uric acid, which substance he looks upon as the primary agent in producing attacks of headache and mental dulness and depression, and even epilepsy. The diet he recommends would be 10 ounces of bread per diem, 12 ounces of vegetables and fruit, and about two ounces each of oatmeal, rice, milk, and cheese. Macaroni, potatoes, &c., can be taken in proportion to the wants of hunger and labour; and for those who have been accustomed to eat much meat, small quantities of protene and glutene could be added. There can be no doubt of the great value of a non-meaty diet in the treatment of epilepsy, but we hesitate to call it a non-nitrogenous one.

